# A Review on the Modification of Polysaccharide Through Graft Copolymerization for Various Potential Applications

**DOI:** 10.2174/1874104501711010109

**Published:** 2017-09-26

**Authors:** Deepak Kumar, Jyoti Pandey, Vinit Raj, Pramendra Kumar

**Affiliations:** 1 Department of Applied Chemistry, MJP Rohilkhand University, Bareilly – 243006(U.P.), India; 2 Department of Applied Chemistry, Babasaheb Bhimrao Ambedkar University, Vidya Vihar, Rae Bareli Road, Lucknow 226025, India; 3 Department of Pharmaceutical Sciences, Babasaheb Bhimrao Ambedkar University, Vidya Vihar, Rae Bareli Road, Lucknow 226025, India

**Keywords:** Polysaccharides, Grafting, Copolymer, Initiator

## Abstract

**Introduction:**

Graft copolymerization is one of the most promising technique uses to modify the properties of naturally available polymers with a minimum loss in their native characteristics.

**Methods and Materials:**

Graft copolymerization is a very significant technique to add hybrid properties in backbone of polymers. The grafting generally initiated through the formation of free radical centers on the polymer backbone as well as monomer.

**Results:**

Grafted polysaccharides have various applications in different important scientific areas such as drug delivery, pharmaceutical field, plastic industry, waste water treatment, tannery effluent treatment, textile industry, agriculture area, *etc*. all of this fascinated us to summarize the major research articles over the last two decades outlining different methods of grafting, surface modification, graft copolymerization of synthetic and natural polymers.

**Conclusion:**

Various redox initiator systems *viz. * Ceric ammonium nitrate, per sulfate, Irradiation, FAS-H_2_O_2_ *etc*. is also explored for grafting of vinyl through conventional and non-conventional techniques.

## 1 INTRODUCTION

Polysaccharides have plentiful abundance in forest, plants, trees, sea shells and microbial resources, either from of exudates, seed or agricultural crops. Some useful polysaccharides can be produced by biotechnical routes [1]. Natural polysaccharides and their derivatives are very useful in controlled release of drug in pharmaceutical and biomedical fields. These are advantageous for controlled drug delivery systems, particularly for prolonged time release of drug and enhancement of the activity of labile drugs due to their protection against hostile environments. 

The polysaccharides hold an advantage over the synthetic polymers because of non-toxicity less cost, biodegradability, and easy availability to their synthetic counterparts. Polysaccharides appear as a very appealing alternative of substitution, because they are renewable raw materials [2]. Natural polysaccharides possess many unique properties, but due some shortcoming simultaneously, particularly shelf life and prone to bacterial attack have limited scope as long-lasting materials. Some synthetic polymers are biodegradable and can be tailor-made easily. Therefore, by composing the individual advantages of polysaccharide and synthetic polymers better shelf life and biodegradability can be achieved. Starch-based derivatives completely biodegradable polymers and have potential applications in biomedical and environmental fields [3]. Polysaccharides, proteins and nucleic acids are basic components in living organic systems. Therefore biopolymers mimicking synthetic polymers have great scope in current and future research to meet out the industrial and scientific requirements. The synthetic polymers can be divided into different categories based on their chemical properties. Some special types of polymers have high category as a very useful class of polymers and have their own special chemical properties and applications in various areas. These polymers are coined with different names based on their physical or chemical properties like stimuli-responsive polymers [4]. Grafting of synthetic polymers onto natural polymers has invited great interest of many researchers because of their applications to biomedical and bio-degradable materials. It is one of the easy methods to increase the compatibility between synthetic polymers and natural polymer. Starch and starch derivatives are used as recipients for tab letting and matrices for delivery systems in the pharmaceutical industry because of biocompatibility, degradability and low costs [5]. Graft copolymerization is a significant technique to add the advanced properties of backbone polymers. It is a chemical technique which modifies the desired features in natural fiber without affecting their inherent behavior. Many researchers have carried out the grafting onto the different polysaccharide backbone using various vinyl monomers, and using a wide range of initiator, oxidizing agent, monomers, binary vinyl monomeric mixtures and radiation techniques and achieved fruitful results [6, 7]. One of the most effective ways of modification of psyllium is by graft copolymerization with suitable monomers [8-10], and the properties of the grafted product can be suitably modulated in terms of percentage grafting. The end product macromolecules are thus tailored at the molecular level for desired applications [11]. Graft copolymerization of vinyl monomers is a universal, effective and accessible method of chemical modification in grant molecules in natural polymer. Considerable work is done on graft co- polymerization of natural polysaccharide such as starch, chitosan, cellulose *etc*. with the vinyl monomers [12]. 

## 2 METHODS AND MATERIALS

Copolymerization is done to improve the properties and the utility of a system in various applications. It allows the synthesis of almost unlimited amount of different products by variation in nature and relative amount of the monomer units in the copolymer. Copolymerization modifies the symmetry of the polymeric chain and modulates both intramolecular forces and properties such as glass transition temperature, crystallinity, solubility, elasticity, permeability and chemical reactivity can be tuned within wide limits. Graft copolymers are prepared by polymerizing a monomer in the presence of a polymer of different backbone chain. Grafting results into retention of desirable properties of base polymer and incorporation of favorable properties in grafted polymer. 

Graft copolymer synthesis is important for the development of polymer science with potential uses in areas such as composites, medical applications, fiber modifications *etc*. Product characterizations are vital in developing structure property relationships. Radical polymerizations is a useful method for the polymerization of a wide variety of vinyl monomers and can be plagued by a lack of control over the mechanism, radical polymerizations have many different reactions occurring simultaneously namely initiation, propagation, termination by coupling, disproportionate or chain transfer. Extending the versatility of radical polymerizations and radical graft copolymerization have been successful in terms of obtaining a grafted derivative, however grafted product characterizations are being inherently difficult as it is further complicated by homopolymer which is amply generated during the reaction [13]. The main aim of this review is to survey the literature on polysaccharides particularly focused on its graft copolymer methodsby using the different initiator and their applications in drug delivery, pharmaceutical field, plastic industry, waste water treatment, tannery waste water treatment, and textile industry. 

Graft copolymers are biodegradable with advanced property and are used in agriculture, textile, paper industry, medical treatment and in the petroleum industry as flocculants and thickening agents. Graft copolymerization can be done with or without the presence of different initiator systems by conventional and non-conventional methods [14]. 

### 2.1 Different Systems of Initiation

Graft copolymerization can be obtained by different initiator systems among them azobisisobutyronitrile (ABIN), ammonium persulfate, potassium persulfate (KPS), ceric ammonium nitrate (CAN), and benzoyl peroxide, ceric ammonium nitrate is widely used for the synthesis of graft copolymers [14]. Bhattacharya *et al. * (2004) investigated that the irradiation of polymer can cause hemolytic fission and thus forms free radicals on the polymer backbone. The medium is necessarily in the radiation technique, *e.g.* if irradiation is carried out in the air, peroxides may be formed in the polymer shown in Fig. (**1**). However, the life of the free radical depends upon the nature of the polymer. 

A graft copolymer is a macromolecular chain with one or more species of block connected to the main chain as side chain(s). Thus, it can be described as having the general structure, where the main polymer backbone, commonly referred to as the trunk polymer, has branches of another polymeric chain emanating from different points along its length. Various methods of graft copolymerization have been reported in the literature [15-20]. 

#### 2.1.1 Different System /Mode of Initiation

##### 2.1.1.1 Ceric Ion Initiation

Nayak *et al.* investigated that ceric ion-induced redox initiation method has been preferred for grafting polymerization because the redox process initiates free radical sites exclusively on the polysaccharide backbone, which reduces the homopolymerization of participating monomers [21]. 

Cerium is a member of the group IIIA of the periodic table. Cerium is tetravalent atom, and has two common oxidation states +3 Ce(III) and +4 Ce(IV). 

Ce(IV)4+e−+→Ce(III)3+

In most of the homogeneous oxidation of substrate by Ce^+4^(IV), the formation of the intermediate complex has been found to be an important prerequisite. Typically ceric ion initiation is performed under acidic aqueous conditions. Acid concentration has affected the rate of polymerization initiated by the ceric ion, but the relationship is empirical. For instance, the following equilibrium is shown in the following equation which is observed in aqueous per chlorate acid solutions, where the ceric ion concentration is dependent upon the acid concentration. 

Ce(IV)4++ 3H2O⇌Ce(OH)3 + 3H+

Generally aqueous ceric ion initiations are performed under acidic conditions to promote higher concentration of Ce(IV) [22, 23]. Sadeghi *et al.*, studied on polymethacrylamide grafted onto carboxymethyl cellulose (CMC) backbone in a homogeneous solution using a ceric ammonium nitrate (CAN) as an initiator and water as solvent [24]. 

**Fig. (2):**
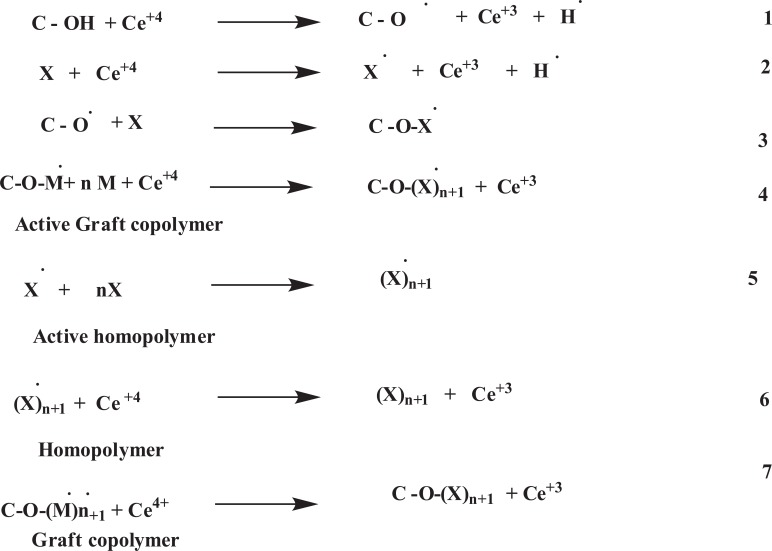
Bioremediation of natural fiber by graft copolymerization.

Chauhan A. *et al.* 2013 synthesized Bioremediation of Natural Fiber by Graft Copolymerization. And also studied the Sorrel stem fiber was graft copolymerized by vinyl monomeric mixtures that lead to an increase in the percentage grafting with a significant change in physico-chemico-thermal resistance. Miscibility in organic solvents, hydrophobicity was found to increase whereas crystallinity, crystallinity index, dye-uptake and hydrophylicity decreased after graft copolymerization. According to Chauhan A. *et al.* grafting of vinyl monomer onto the polymeric backbone occurred as follows (Fig. **2**) [25]. 

Where, C-OH = sabdariffabackbone and M = monomer

Ikhuoria *et al.*, synthesized graft copolymerization of acrylonitrile onto cassava starch by ceric ion induced initiation [26]. Rate evolution of binary grafting polymerization of buthylmethacrylate and acrylic acid onto carboxymethylcellulose by ceric ion induced initiation studied by Sadeghi *et al. * [27]. Sekhar *et al.*, investigated chitosan and guar gum-g-acrylamide semi interpenetrating microspheres (semi IPNMs) were prepared by water-in-oil emulsion cross linking method using glutaraldehyde as a crosslinker. 5-fluorouracil (5-FU) is an anticancer drug was successfully loaded in these semi IPNMs. X-ray diffraction (XRD) and differential scanning calorimetric (DSC) examined the crystalline nature of drug after encapsulation into semi IPNMs. Scanning electron microscopy (SEM) reveals the formation of semi IPNMs is spherical with size around 200 µm. The encapsulation efficiency of 5-FU was achieved up to 58%,in-vitro release studies were performed basic (pH 7. 4) buffer medium [28]. Banyal *et al.*, synthesized Grafting of binary mixtures of methyl methacrylate and some vinyl monomers onto mulberry silk fiber. Synthesis, characterization and preliminary investigations into gentian violet uptake by graft copolymers by ceric ion induced initiation [29]. Dholakia *et al.*, studied grafting of acrylonitrile (AN) onto sodium salt of partially carboxymethylated psyllium (Na-PCMPsy) has been carried out using a ceric ammonium nitrate (CAN) as a photo initiator in an aqueous medium [27]. Pati *et al.*, synthesized graft copolymerization of methyl methacrylate onto chitosan was investigated using ceric ammonium nitrate as the initiator. The effects of initiator concentration, monomer concentration, time and temperature on %G and %GE were studied [30]. Dincer*et al.*, investigated the polymerization of acrylamide, initiated by a cerium (IV) ammonium nitrate-methionine redox initiator system was carried out in an aqueous solution at different reaction conditions [31]. Natural fibers have received vast attention because of their combustible, non-toxic, low cost, hydrophilic and biodegradable properties. In this study, functionalization of cannabis indica fiber has been carried out by its grafting with acrylic acid (AAc) using Ce (+3)/ HNO_3_ redox initiator couple in aqueous medium [32]. 

##### 2.1.1.2 Persulphate Initiation

**Fig. (3):**
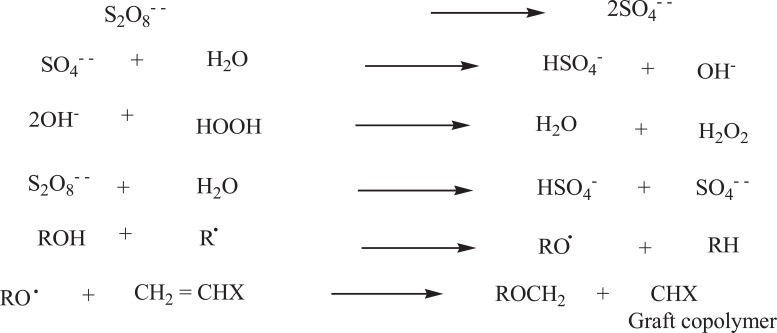
General reaction mechanism graft copolymerization by persulphate initiation.

Generally ammonium persulphate, potassium persulphate or ferrous persulphate are used as initiators for graft polymerization. When an aqueous solution of persulphate is heated it decomposes to yield sulphate radical along with free radical species. The mechanism for grafting, postulated [33] as follows (Fig. **3**):

Where R-OH = polysaccharide, R Free radical species. 

We investigated from the literature that with anincrease in the concentration of persulfate initiator, percentage grafting and efficiency grafting initially increased,but with further increase in [persulfate initiator] beyond certain limit, these grafting parameters showeddecreasing tendency. This behaviour was explained by the fact that with the increase in [persulfate initiator] there is concurrent increase in free radical formation which is able to attack polysaccharide chain of GG/its derivatives.

This results in the formation of more macro radicals capable of grafting vinyl monomers on them. On further increasing the [persulfate initiator] above the thresholdvalue equivalent to maximum grafting, large number of free radicals are formed which may initiate homopolymerization of vinyl monomers at the expense of grafting. 

**Fig. (4):**
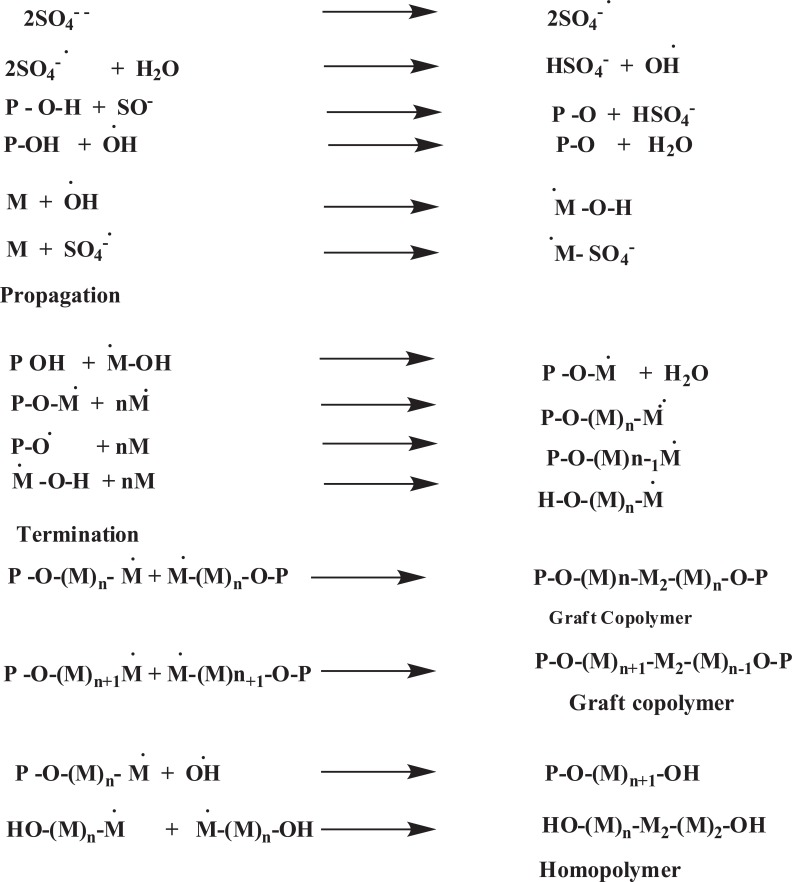
Synthesis of Cross-linked Psy-*g*-poly(AA).

Singh *et al.*, synthesized psyllium and polyacrylamide based hydrogels for the use in drug delivery they had prepared psyllium and polyacrylamide based polymeric networks by using N,N-methylenebisacrylamide (N,N-MBAAm) as crosslinker [34]. Psyllium is a medicinally active natural polysaccharide, had been modified with polyacrylamide to develop the hydrogels, those can act as the potential candidate for novel drug delivery systems [35]. Kumar *et al.*, studied psyllium and acrylic acid based polymeric networks were synthesized under different reaction conditions such as either air or inert conditions, and under the influence of γ-radiations using potassium persulphate (KPS) & hexamethylenetetramine(HMTA) as an initiator-crosslinker system [36]. Prashar *et al.*, synthesized gumtragacanth-acrylic acid based hydrogels using KPS-ascorbic acid and glutaraldehyde as an initiator-crosslinker via free radical polymerization technique was also carried out [37]. Kaith *et al.*, studied psyllium mucilage which was obtained from plantagoovatahad been modified through graft copolymerization and network formation using acrylic acid (AA) as the monomer, potassium persulphate (KPS) as an initiator and hexamethylenetetramine (HMTA) as cross-linker. The mechanism of this copolymerization reaction is shown in (Fig. **4**) [38]. 

M = monomer free radical Psy-O*= Backbone free radical (psyllium) [38]. 

Kaith *et al.*, investigated psyllium had been functionalized with acrylamide in the presence of KPS/HMTA couple as an initiator-crosslinker system [39]. 

##### 2.1.1.3 Irradiation

Nagger *et al.*, reported hydrogels based on tara gum/acrylic acid (TG/AAc) were prepared by gamma irradiation, in the presence of N,N-methylene-bis-acrylamide (MBAAm) as a crosslinking agent [40]. Psyllium-N-vinylpyrrolidone based hydrogels by radiation induced crosslinking are also reported. Polymers were characterized with SEM, FTIR and swelling studies. Swelling of the hydrogels was studied as a function of monomer concentration, total radiation dose, temperature, pH and salt taken by the swelling medium [39]. Polymeric flocculent has been developed by graft copolymerization of acrylamide (AAm) with acrylic acid (AAc) using gamma irradiation technique. The grafted copolymer (PAAm/AAc) was characterized using fourier-transform infrared spectroscopy (FTIR), and thermo-gravimetric analysis (TGA). The effects of reaction parameters such as total absorbed dose and monomer concentration on grafting yield were investigated [41]. Iskandar synthesized the graft copolymer of methyl methacrylate onto starch and natural rubber latex was conducted by a simultaneous irradiation technique. Gamma rays from cobalt-60 source were used as the initiator [42]. Shanmugapriya *et al.*, studied graft copolymer of chitosan with acrylic acid polymer has been synthesized using ceric ammonium nitrate and nitric acid redox system under UV irradiation [43]. 

##### 2.1.1.4 FAS - H_2_O_2_ as Redox Initiator

**Fig. (5):**
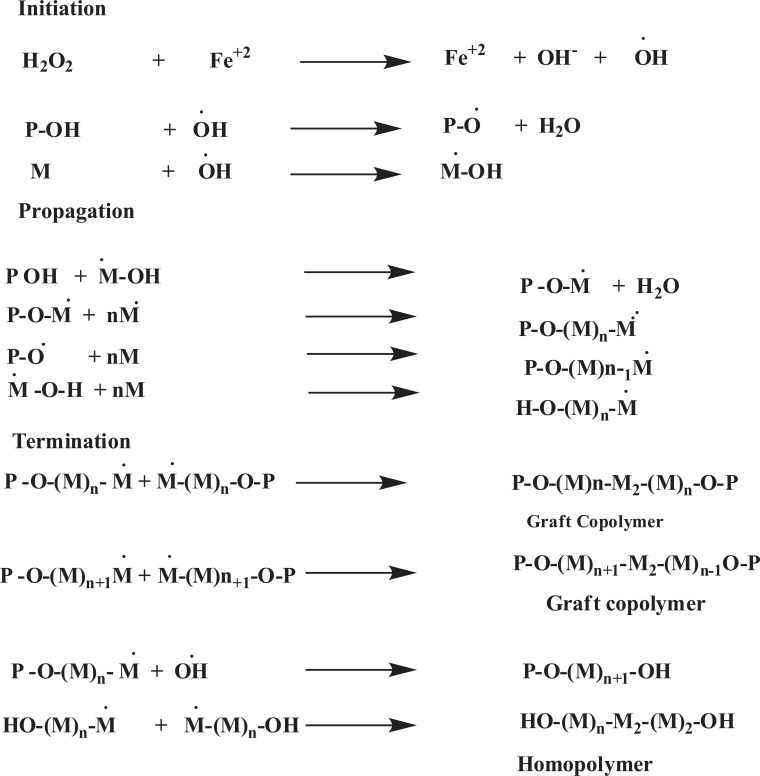
Psy-g-poly(AA) hydrogel.

Kalia *et al.*, synthesized a medicinally important natural psyllium polymer was functionalized with acrylic acid using FAS-H_2_O_2_ as a redox initiator and glutaraldehyde as a crosslinker. Schematically the reaction mechanism of the graft copolymerization of monomer onto polymer illustrated in (Fig. **5**) [44].

##### 2.1.1.5 Microwave Energy Induced Initiation

**Fig. (6):**
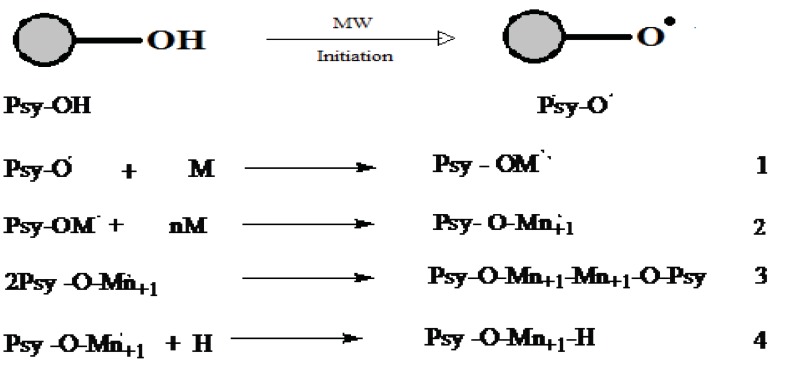
Mechanism for “microwave-initiated synthesis of Psy-g-PAM”. Psy-OH = Psyllium, MW = Microwave Radiation, M = Monomer (Acrylamide).

The use of microwave energy has been used in the past two decades to improve the procedural limitation in the synthesis of a range of graft modified polysaccharide material. In fact, the increasing interest in green and clean environment friendly chemistry has motivated the use of microwaves in the polysaccharide grafting modification. Microwave irradiation significantly reduced the use of toxic solvents, as well as the reaction time in mostly the grafting reaction of interest here, ensuring high yields product selectivity and cleans product formations. Furthermore, in many instances microwave synthesized polysaccharide copolymer reveal better properties for commercial exploitation than their conventionally synthesized counterparts [45]. In this way, the relatively higher yields and grafting efficiency could be achieved within a very short time with no or little addition of any radical initiators or catalyst, and the extent of grafting could be adjusted by controlling the microwave conditions [46-48]. Microwave radiations cause “selective excitation” of the polar bonds only, which in turn leads to their rupture/cleavage. This cleavage of bonds creates many free radical sites on the polymer backbone. The “C-C” sequence of the backbone polymer remains unaltered by the microwave radiation since it is relatively nonpolar. The reaction follows the mechanistic pathway as shown in (Fig. **6**) [49]. 

It depicts the graft polymerization mechanism initiated by individual microwave and based on free radical mechanism. Typically, the polar O-H bond can easily be broken under the action of microwave radiation, while the C-C bond (practically nonpolar) has not been affected. The cleavage of the O-H bonds leads to the formation of free radical “active” sites on the backbone of psyllium. These active sites can react with vinyl monomers to achieve the growth of chains, and the graft copolymer could be formed [50, 51]

### 2.2 Graft Copolymerizationof Polysaccharides Using Different Monomers

Among various methods graft copolymerization is the most attractive because it is a useful technique for modifying the chemical and physical properties of natural polymers [52, 53]. 

#### 2.2.1 Grafting by Acrylic Acid

Psyllium and acrylic acid based polymeric networks under different reaction conditions such as in air, under inert condition and under the influence of radiations using potassium persulphate (KPS)-hexamethylenetetramine (HMTA) as an initiator-crosslinker system [54]. Banyal *et al. * reported that mulberry silk fiber was graft copolymerized with binary mixtures of acrylic acid, methyl acrylate and acrylonitrile with methyl methacrylate as the principal monomer in aqueous medium by using CAN as redox initiator. The binary vinyl monomers were graft copolymerized by using the grafting conditions like reaction time, temperature, concentration of MMA and CAN as reported earlier in optimum percent grafting of MMA alone onto the same backbone. Graft copolymers were characterized by FTIR, SEM, swelling studies, moisture absorption and chemical resistance in acidic and alkaline medium. Dye uptake (gentian violet) on graft copolymers were studied photo-calorimetrically at 420 nm. The dying capability of the graft copolymers with binary mixture is more than the reference graft copolymer of methyl methacrylate [27]. Hydrogels based on tara gum/acrylic acid (TG/AAc) were prepared by gamma irradiation, in the presence of N,N-methylenebisacrylamide (MBAAm) as a crosslinking agent. The polymeric networksformed were characterized by FTIR and evaluated by swelling studies as a function of MBAAm concentration, temperature and nature of the swelling medium. The swelling kinetics of the hydrogels was studiedin terms of the diffusion exponent. The results showed that the water diffusion into hydrogels is anon-fickian type. Stress-strain curves of hydrogels were evaluated to calculatethe shear modulus values and the average molecular weight between crosslinks. Moreover,the absorption under load at 37^0^C of water and urea aqueousby TG/AAc hydrogels was determined [48]. Singh V. *et al.* (2010) studied, efficient mercury sorbent had been crafted by poly(acrylic acid) grafting onto C. javanica seed gum under microwave irradiation and synthesised graftcopolymer samples withvarying the reaction conditions of different %G . Environment is deteriorating day by day due to industrial pollution, toxic chemical leads to the accumulation of heavy metals contamination in the waste water. The waste water polluted by these effluents disturbs the normal use of water for agriculture & aquatic life. The purity and quality of water is a basic concern for mankind. Mostly coal products synthesis in water medium and water content of coal has a negative impact on handling and specific energy value. The moisture content may be attributed to the proportion of fine coal, which presents the greatest dewatering problem. A novel polymeric flocculant has been developed by graft copolymerization of acrylamide (AAm) with acrylic acid (AAc) using gamma irradiation technique [55]. 

Natural polymers are industrially attractive because of their capability of removing the metal ions present in the waste water. Among the many other low cost absorbents identified chitosan had the highest sorption capacity for several metal ions [56]. The use of biosorbent chitosan makes it possible to remove both heavy metals and organic compounds. Chitin,chitosan, grafting acrylic acid polymers are individually used for waste water treatment due to biocompatibility and low-cost. Deshpande *et al.*, has synthesized graft copolymers of chitosan with acrylic acid polymer had been synthesized using ceric ammonium nitrate, nitric acid redox system under UV irradiation [57]. 

Kumar *et al. * synthesiszed psyllium and acrylic acid based polymeric networks under different reaction conditions such as in air, in vacuum and under the influence of γ-radiations using potassium persulphate (KPS) hexamethylenetetramine (HMTA) as an initiator-crosslinker system. Initially, optimization of various reaction parameters was performed under all the different reaction conditions. It had been observed that the percent grafting varies with varying reaction conditions with maximum grafting (61. 15%) reported in case of synthesis carried out invacuum followed by the synthesis in air (61. 00%) and then in case of synthesis under the influence of γradiations [58]. Graft copolymerization of butylmethacrylate (BMC) and acrylic acid (AA) onto carboxymethylcellulose (CMC) was carried out under argon atmosphere in a homogeneous aqueous medium by using ceric ammonium nitrate (CAN) as an initiator [23]. 

Kaith *et al.*, observed psyllium mucilage which was obtained from plantagoovata hasbeen modified through graft copolymerization and network formation using acrylic acid (AA) as the monomer, potassium persulphate (KPS) as an initiator, and hexamethylenetetramine (HMTA) as a cross-linker [59]. 

#### 2.2.2 Grafting by Acrylo-Nitrile

The copolymer of plantago psyllium mucilage and acrylonitrile had beensynthesized in the presence of nitrogen using ceric ion/nitric acid redox couple. Polyacrylonitrilegrafted Psyllium(PSY-g-PAN) was characterized by IR spectroscopy and tested for its flocculation efficiency in textile effluent by the standard jar test method. The efficiency of removal of solid waste from textile effluents depends various parameters viz. adsorbent dose, temperature, pH, contact time, RPM *etc* [60]. Photo induced grafting of acrylonitrile (AN) onto sodium salt of partially carboxymethylated psyllium (Na-PCMPsy) had been carried out using ceric ammonium nitrate (CAN) as a photo initiator in aqueous medium. The reaction variables including concentrations of initiator, nitric acid, monomer and amount of the backbone as well as time and temperature have been varied for establishing grafting. The influence of these reactions conditions on the grafting yield hadbeen discussed. The FTIR spectra, thermal analysis (TGA) and scanning electron microscopic (SEM) techniques had been used for the characterization of graft copolymer [29]. Plantago psyllium mucilage (PSY), an anionic natural polysaccharide consisting of pentosan and uronic acid obtained from the seeds of plantago psyllium (*Plantago family*), was grafted with acrylonitrile (AN). Graft copolymers were prepared by ceric ion initiated solution polymerization technique and was characterized by FT-IR spectroscopy, scanning electron microscopy and differential scanning calorimetric [61]. Photo-induced grafting of acrylonitrile (AN) onto sodium salt of partially carboxymethylated psyllium (Na-PCMPsy) carried out using ceric ammonium nitrate (CAN) as photoinitiator in an aqueous medium. The reaction variables including concentrations of initiator, nitric acid, monomer and amount of the backbone as well as time and temperature had been varied for establishing grafting [19]. V. *et al. * (2005), prepared the graft copolymer of Cassia siamea with acrylonitrile under microwave (MW) irradiation without adding any radical initiator or catalyst. Freeradicals are generated here due to the dielectric heatingcaused by the localized rotation of the hydroxyl groups atthe polysaccharide backbone and initiate grafting. Graft copolymerization done by following reaction mechanism and observed that microwave promoted grafting over the conventionalgrafting, %*G* and %*E* for both the methods were compared [62]. Vandna *et al. * 2003 prepared the grafting of polyacrylonitrile (PAN) onto guar gum in water, without using any radical initiator or catalyst within a veryshort reaction time through the microwave (MW) irradiation [63]. 

Ikhuoria*et al.*, studied on the graft copolymerization of acrylonitrile onto cassava starch by ceric ion induced initiation the graft copolymerization. Ten grades of graft copolymers were synthesized five by varying the initial concentration of the monomer and the other five by varying the initial concentration of the initiator. Evidence of graft copolymerization of the hydrolyzed products was obtained from the air analyses. Some grafting parameters such as % grafting ratio and % conversion were favoured by initial increase in the monomer concentration. However, these parameters were observed to decrease at much higher concentrations (>3 M). Evidence of hydrolysis shows that the grafted copolymers could be used as flocculants [24]. Singh V *et.al* 2007, have synthesised Starch-g-poly(acrylonitrile)Using a very low concentration of potassium persulfate as initiator, acrylamide could be efficiently grafted onto starch under microwave irradiation and for the grafting O_2_ removal from the reaction vessel was not required [64]. 

Mishra *et al.*, synthesized acrylonitrile grafted Plantago psyllium in the presence of nitrogen using ceric ion-nitric acid redox system. The effects of polymer dose, pH and contact time on the removal of solid waste from textile effluent are reported. The optimum dose was found to be 1.6 mg/L, at which a maximum solid removal of 94% suspended solid (SS) and 80% total dissolved solid (TDS) was seen. The most suitable pH was acidic (pH 4.0) and neutral (pH 7.0), for SS and TDS removal, respectively. The optimum treatment duration was one hour. X-ray analysis of PSY-g- PAN and solid waste from effluent before and after treatment suggests the interaction of the solid waste with the PSY-g-PAN copolymer [9]. Singh *et al.*, reported, graft co-polymerization of acrylonitrile (AN) onto saccharumcilliarefibre has been carried out in the presence of potassium persulphate and ferrous ammonium sulphate (FAS-KPS) as redox initiator in an autoclave. Various reaction parameters such as pressure, time, pH, concentrations of initiator and monomer were optimized to get maximum graft yield (35·59%). Grafted and ungrafted saccharincilliare fibres were then subjected to evaluation of some of their properties like swelling behavior in different solvents, moisture absorbance under different humidity levels, water uptake and resistance towards chemicals such as hydrochloric acid and sodium hydroxide. The characterization of the graft copolymers were carried out by FTIR spectrophotometer, X-ray diffraction (XRD) and scanning electron microscopic (SEM) studies [65]. Polyacrylonitrile grafted agar/ sodium alginate had been synthesized in aqueous medium under reflex condition in the presence of potassiumpursulfate as afree radical initiator [66]. 

#### 2.2.3 Grafting by Acryl Amide

Most coal preparations are carried out in water medium and moisture and water content are problematic in handling and specific energy value. A novel polymeric flocculants had been developed by graft copolymerization of acrylamide (AAm) with acrylic acid (AA) using γirradiation technique [41]. Saifuddin and Yusumira observed that polymers had been molecularly imprinted for the purpose of binding specifically to α-tocotrienol (vitamin E). A molecularly imprinted polymer (MIP) material was prepared using α -tocotrienol as the imprinted molecule, acrylamide as functional monomer and macro porous chitosan beads as functional matrix. Chitosan-graft-polyacrylamide was synthesized without any radical initiator or catalyst using microwave (MW) irradiation [67]. Ahuja *et al. * 2009 have synthesized Xanthan-g-poly(acrylamide) under the microwave radation. Xanthan-g-poly(acrylamide) was prepared employing microwave-assisted grafting and ceric-induced grafting. Microwave assistedgrafting of acrylamide on xanthan gum was done using themethod reported earlier [68]. 

Hossein Hosseinzadeh reported the effect of different reaction conditions on the grafting of acrylamide (AM) onto k-carrageenan (kC) using potassium persulfate (KPS) initiator had been studied by determining the grafting parameters. The reactions were carried out under argon atmosphere in a homogenous aqueous medium; the graft copolymer was characterized by FTIR spectroscopy. It was observed that with increasing AM, kC, and KPS concentrations as well as reaction time and temperature the grafting parameters were also increased, but further increase of reaction conditions disfavored these parameters [69]. Vandna *et al. * (2005). Synthesized Chitosan-graft-polyacrylamide (Ch-g-PAM) without any radical initiator or catalyst using microwave (MW) irradiation. Under optimal grafting conditions, 169% grafting was observed at 80% MW power in 1. 16 min [70]. 

**Fig. (7):**
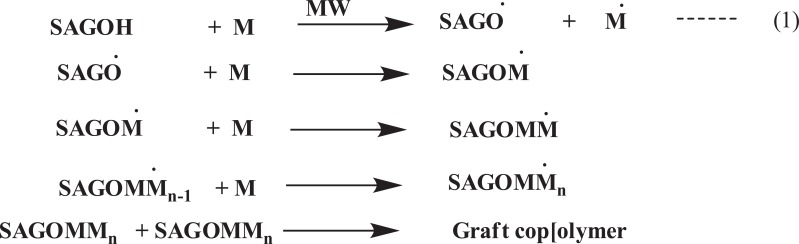
Synthesis of SAG-g-PAM from SAG using microwave irradiation.

Polyacrylamide grafted cellulose had also been demonstrated to be a very efficient and selective sorbent for removal of mercuric ions from synthetic aqueous solutions. The mercury-uptake capacity of the graft polymer is as high as 3. 55 mmol/g and sorption is also reasonably prompt. Thus, 0. 2 g of the graft copolymer is able to extract 50 ppm Hg(II) from 50 ml water completely in less than ten minutes. The Hg (II) sorption is selective and no interferences have been observed in the presence of Ni (II), Co (II), Cd(II), Fe(III), Zn(II) ions in 0. 1 M concentrations at pH 6. Regeneration of the loaded polymer without losing its original activity can be achieved using hot acetic acid. The graft copolymer described seems very suitable for removal of large amounts of mercury in hydrometallurgical applications and may also be useful for other water treatments [71]. Pal *et al.* (2010) synthesised the polyacrylamide-grafted sodium alginate, through microwave radiation and investigated the effect of reaction parameters (*i.e.*, irradiationtime and monomer concentration) onto the percentage grafting. Microwave irradiation was used to generate the free-radical sites on the polysaccharide backbone. The reaction follows the mechanistic pathway as shown in (Fig. **7**) [72]. 

Singh V *et al.* (2006), have synthesised PotatoStarch-g-poly(acrylamide) using a very low concentration of potassium persulfate as initiator, acrylamide could beefficiently grafted onto potato starch under microwave irradiation and for the grafting O_2_ removal from the reaction vessel was not required [73]. 

#### 2.2.4 Polymermethylmethaacrylategrafting

UV-radiation induced grafting of methyl-methacrylate onto sodium salt of partially carboxymethylated psyllium had been carried out using ceric ammonium nitrate as a photo initiator in an aqueous medium. The reaction variables including concentrations of initiator, nitric acid, monomer and amount of the backbone as well as time and temperature have been varied for establishing the optimized reaction conditions for grafting. Sadeghi *et al.*, reported grafted polymethacrylamide (PMMA) onto Carboxymethyl cellulose (CMC) backbone in a homogeneous solution using ceric ammonium nitrate(CAN) as an initiator and water as solvent. The structure of virgin CMC sample and grafted with monomers was characterized by FTIR spectra and TGA analysis. The thermal properties of pure CMC and grafted with monomers were evaluated with a simultaneous thermal analysis system. The results showed that the thermal stability of grafted samples was appreciable improved. The effects of various reaction conditions such as monomer, polyssacharide, initiator concentration and reaction temperature on the percentage of conversion, graft yield (G %) and graft efficiency (GE %) were investigated [27]. 

The graft copolymerization of methyl methacrylate onto starch and natural rubber latex was conducted by a simultaneous irradiation technique to obtain the degradable plastic γ-rays from cobalt-60 source was used as the initiator. The grafted copolymer of starch-polymethyl methacrylate and the grafted copolymer of natural rubber-polymethyl methacrylate were mixed in the blender, and dried it in the oven. The dried grafted copolymer mixture was then molded using hydraulic press machine. The effect of irradiation dose, composition of the grafted copolymer mixture, film forming condition and recycle effect was evaluated. The parameters observed were tensile strength, gel fraction and soil burial degradability of grafted copolymer mixture. It was found that the tensile strength of grafted copolymer mixture increased by γ-ray irradiation. Increasing of the grafted copolymer of natural rubber-polymethyl methacrylate content, the gel fraction and tensile strength of the grafted copolymer mixture increased by Iskandar [43]. The graft copolymerization of methyl methacrylate onto chitosan was investigated using ceric ammonium nitrate as the initiator. The effect of initiator concentration, monomer concentration, time and temperature on %G and %GE were studied. The antibacterial activity of chitosan as well as the grafted samples was investigated using some gram positive and gram negative bacteria. Grafted products improved considerably the antibacterial activity [31]. The UV-radiation induced grafting of methyl-methacrylate onto sodium salt of partially carboxymethylated psyllium carried out using ceric ammonium nitrate as a photoinitiator in an aqueous medium. The reaction variables, including concentrations of initiator, nitric acid, monomer, and amount of the backbone as well as time and temperature had been varied for establishing the optimized reaction conditions for grafting. The influence of these reaction conditions on the grafting yields had been discussed [74]. Singh V. *et al. * prepared chitosan-graf-poly methylmethacrylate by using the microwave radiation by following way Chitosan dissolved in 25 ml of 5% aqueous formic acid and methylmethacrylate (17x10^-2^M) was irradiated in adomestic microwave oven in a 150 ml flask. Chitosan-graft –poly (methylmethacrylate) (Ch-g -PMMA) was separated from poly methymethacrylate [75]. Babu and Dhamodharan reported that polymethylmethacrylate (PMMA) in the brush form is grown from the surface of magnetite nanoparticles by ambient temperature atom transfer radical polymerization (ATATRP) using a phosphonic acid based initiator. The surface initiator was prepared by the reaction of ethylene glycol with 2-bromoisobutyrl bromide, followed by the reaction with phosphorus oxychloride and hydrolysis. This initiative is anchored to magnetite nanoparticles via physisorption. The ATATRP of methyl methacrylate was carried out in the presence of CuBr/PMDETA complex, without a sacrificial initiator, and the grafting density was found to be as high as 0. 90 molecules/nm^2^. The organic–inorganic hybrid material thus prepared shows exceptional stability in organic solvents unlike unfunctionalized magnetite nanoparticles which tend to flocculate. The polymer brushes of various number average molecular weights were prepared and the molecular weight was determined using size exclusion chromatography, after degrading the polymer from the magnetite core [76]. 

## 3 RESULTS

### 3.1 Applications Grafted Copolymer

#### 3.1.1 In Pharmaceutical Field

A wide variety of grafted natural polysaccharides had been used to fabricate different types of drug delivery system. Natural polysaccharides and their derivatives were used to controlled release of drug in pharmaceutical and biomedical field. The advantages of controlled drug delivery system are mainly the achievement of an optimum concentration, usually for prolonged times, the enhancement of the activity of labile drugs due to their protection against hostile environments and the diminishing of side effects due to the reduction of high initial blood concentration [2]. The antibacterial activity of chitosan as well as the grafted samples was investigated using some gram positive and gram negative bacteria. Grafted products improved considerably the antibacterial activity [29]. Physiological substances play very importantrole in food due to their ability to deactivate free radicals in organisms. Free radicals may bea danger to cells and their components if their level of concentration was not controlled. Insuch a case, cancer and other severe diseases may occur. The control of the radicals isrealized via the activity of antioxidants like Vitamins A, C or E1 which may donate electronsto the radicals. Vitamin E is one of the most important lipid-soluble primary defense antioxidants. Vitamin E is a potent antioxidant that protects the body against oxidative damage, notably cell membranes and cholesterol transporting lipoproteins [77]. Singh *et al.*, also reported that the tetracycline hydrochloride drug was released from the modified psyllium with polymethacrylamide polymeric networks by using N,N-MBAAm as crosslinker and ammonium persulfate (APS) as initiator which used in colon specific drug delivery. The release of water-soluble drug tetracycline hydrochloride entrapped in ahydrogels occur only after water penetrates the polymeric networks to swell and dissolve the drug followed by diffusion along the aqueous pathways to the surface of the device. The effect of pH on the release pattern of tetracycline was studied in varying the pH of the release medium. However, the amount of drug release in pH 7. 4 buffer solutions was higher than the pH 2. 2 buffer and distilled water [78]. 

#### 3.1.2 In Plastic Industry

Plastic is valuable material for human life and used in vast range of products. Plastic materials are quite significant due to their easy ofprocessing, versatility and imperviousness to water. Polymers have already substuited many traditional materials, such as wood, metal, glass, and ceramic, in most of their former uses [41]. The biggest problem with plastic recycling is difficult to automate the sorting of plastic waste, and so it require intensive labor scientist carried out lots of research on biodegradable plastics. The difficulty can be let down by break down of plastic mateials with exposure to sunlight, water or dampness, bacteria, enzymes, insect attack are also included as forms of biodegradation or environmental degradation. Combination of double biodegradable initiator materials such as starch and natural rubber latex with polymethyl methacrylate was expected to obtain renewable degradable plastic [79, 80]. 

#### 3.1.3 In Waste Water Treatment

The chemical pollution of water from a wide range of toxic pollutants particularly clay metals, aromatic molecules and dyes. These materials have serious environmental problem undischarged to their potency in human toxicity. Therefore, it requires developing technology that can overcome toxic pollutants found in wastewater. Water pollution due to toxic metals and organic intensifies remains a serious environmental and public problems. Moreover, faced with more and more rigorous regulations. Water pollution has also become a major source of concern and an antecedent for most industries. Heavy metal ions, aromatic compounds (including phenolic derivatives, and polycyclic aromatic compounds) and dyes are often found in the environment as a result of their broad industrial uses. They are common contaminants in waste water and many of them are known to be toxic or carcinogenic [81]. Psyllium with acrylic acid based polymeric networks were used for remotion, separation, and enrichment of risky metal ions from aqueous solutions and played a significant role for environmental redress of municipal and industrial waste water [82]. 

#### 3.1.4 In Tannery Waste Water Treatment

Polyacrylamide grafted psyllium mucilage is used as flocculant for treatment in tannery and domestic wastewater, and is was also used for suspended solid removal from tanneries and other wastewater. The flocculation efficiency of the optimal dose of grafted mucilage showed pH for tanneries and domestic waste water respectively. The maximum solid removal was found an alkaline pH (9.2) in the case of tannery effluent and at acidic pH (4.0) in the case of domestic wastewater [83]. 

#### 3.1.5 In Textile Industry

Indian textile industry is widely classified as woolen industry and cotton industry. The industries based on woolen and cotton fibers also include celluloid fiber blends. These industries in general consume large volumes of water of high spinelessness. Consequently, these industries firing large amounts of effluent that normally showing polluting characteristics. The discharge of the effluents to the environment without right treatment may have long lasting consequences. In the last decade, industry and government have become increasingly aware of the need to clean up textile effluents and reduce river pollution. Textile treating plants utilized a wide variety of chemicals such as bases, acids, salts, detergents, sizing oxidants, wetting agents, dyes, and mercerizing and finishing chemicals. Many of these were not retained in the final product and were discharged in the effluent. Without proper discussion the discharge of textile effluent to the environment can cause serious and long-lasting consequences. Plantago psylliummucilage was grafted with acrylonitrile used as flocculant in textile waste water treatment. Removal of suspended solid (SS) and total dissolvedsolid (TDS) was increased in the polymer, the percent removal of solid waste increases, but after a certain dose of polymer, a decreasing trend in solid removal increased with polymer engrossment. The flocculation efficiency of the grafted polymer was found to be the level best at pH 4.0 for SS and pH 7.0 for TDS removal, using its optimum concentration. It was apparent that maximum SS removal (94.4%) takes place over 1 hour of contact time at acidic pH (4.0), neutral pH (7.0), and at alkaline pH (9.2), only 10.5 and 44.3% SS was removed. In the case of TDS, the maximum removal (80.6%) was seen at neutral pH [84].

#### 3.1.6 In Agriculture Areas

In agriculture Pesticides, herbicides, fungicides, and fertilizershave been exploited without considering their disadvantages concerned to the pollution. After being used for at least two to three decades, the world was witnessing the hazardous effects of these chemicals. The properties of pesticides such as volatility, leaching, and photodegradation led to the increased dosage of pesticides. The presence of excess pesticides in soil can prove harmful in many ways. Only a little amount (5-10%) of the applied chemicals was effectively used, the rest being washed away by rain and thereby, reaching the human and aquatic life cycle. Pesticides are one of the major components of water pollution, which can enter living species through the water cycle, thereby disturbing the food chain. These chemicals act on the nervous system, circulatory system and genetic system, and hence creating disorders which may be carried over for generations together. 

## CONCLUSION

Graft copolymerization is a distinctive method among different techniques for modifying natural polymers mostly for polysaccharides. Graft copolymerization is an effective method to incorporate useful properties to the main polymer backbone, and these are useful in many applications in different fields. Grafting of synthetic polymer is an easy method to add new properties to a natural polymer with minimum loss of the initial properties of the substrate. Grafted copolymer plays a vital role in changing its physical-chemical properties. Graft co-polymerization is an efficient means to incorporate the desired feature into polysaccharide. Grafted polysaccharide also more stable than its virgin counterpart. Inthe present review, weare reportingthe syntheses, characterizations and applications of polysaccharide grafted/crosslinked copolymers from the above it can be concluded that the polysaccharide grafted/crosslinked copolymer is an efficient and novel technique and have wide areas of the application such as in drug delivery, adsorption, treatment of textile/tannery, domestic/sewage waste water and also in agriculture and serving for mankind. 

## Figures and Tables

**Fig. (1):**
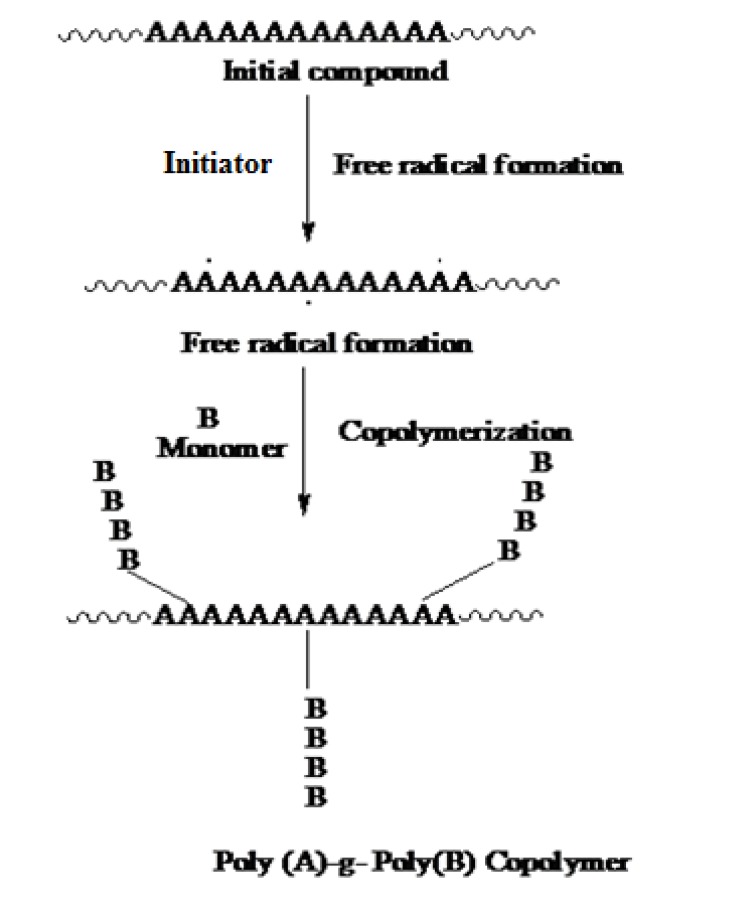
General free radical mechanism of graft copolymerization of vinyl monomer B onto trunk polymer poly (A) to form the graft copolymer poly (A) -*g*-poly (B).
